# Osteoinduction in Novel Micropores of Partially Dissolved and Precipitated Hydroxyapatite Block in Scalp of Young Rats

**DOI:** 10.3390/ma14010196

**Published:** 2021-01-03

**Authors:** Masaru Murata, Jun Hino, Md. Arafat Kabir, Kenji Yokozeki, Michiko Sakamoto, Takehiko Nakajima, Toshiyuki Akazawa

**Affiliations:** 1 Division of Oral Regenerative Medicine, Health Sciences University of Hokkaido, Hokkaido 061-0293, Japan; hino@hoku-iryo-u.ac.jp (J.H.); kabir@hoku-iryo-u.ac.jp (M.A.K.); yokozeki@hoku-iryo-u.ac.jp (K.Y.); 2 HOYA Technosurgical Corporation, Tokyo 160-0004, Japan; michiko.sakamoto@pc.hoya.co.jp (M.S.); takehiko.nakajima@pc.hoya.co.jp (T.N.); 3 Industrial Technology Research Department, Hokkaido Research Organization, Hokkaido 060-0819, Japan; akazawa-toshiyuki@hro.or.jp

**Keywords:** BMP-2, bone, dissolution-precipitation, HAp, micropore, osteoinduction

## Abstract

Osteoinduction in muscles by porous ceramics has been reported to be a real phenomenon. In this study, osteoinduction in connective tissues was found in highly porous hydroxyapatite (HAp) ceramics with large specific surface areas. We have developed the combination method of the partial dissolution-precipitation (PDP) technique involving the stirring-supersonic treatment in 1.7 × 10^−2^ N HNO_3_ solution containing Ca^2+^ and PO_4_^3−^ to improve the surface and the bulk of commercially available synthetic HAp block (82.5% in porosity, 50–300 µm in pore size). The modified HAp was named as a partially dissolved and precipitated HAp (PDP-HAp). The PDP-HAp exhibited the porosities of 85–90%, the macropore sizes of 50–200 µm, and the specific surface areas of 1.0–2.0 m^2^/g, with microcracks. The aim of this study was to observe bone induction by the PDP-HAp with or without BMP-2 in scalp tissues of four-week-old rats. Young rats were divided into the PDP-HAp alone group and the PDP-HAp/BMP-2 group for a long-term observation. In the PDP-HAp group, bone induction occurred inside the many pores at nine months, and the ratio of induced bone was 12.0%. In the PDP-HAp/BMP-2 group, bone induction occurred in almost all pores at three months, and compact bone was found at nine months. The ratios of induced bone were 77.0% at three months and 86.0% at nine months. We believe that osteoinduction by the PDP-HAp might be different from the process of BMP-loaded HAp-induced bone formation, because the PDP-HAp has osteogenic microporous compartments with partially absorbable HAp crystals. The PDP technique may contribute to create bioceramics with osteoinductive property for bone regenerative medicine.

## 1. Introduction

### 1.1. Mechanism of Osteoinductive Process in Microporosity of Ceramics

Porous calcium phosphate scaffolds with microporosity (pore size smaller than 10 μm) induced ectopic bone formation intramuscularly in several animals [1,2,3,4], without the addition of bone morphogenetic proteins (BMPs) [5]. The chemical composition of scaffolds is not the only qualification for osteoinduction. Generally, ectopic bone was found in micropores of ceramics in the muscles of large animals [1,2,3,4,5], and the microporosity played an important role in enhancing the osteoinduction of scaffolds [6,7,8,9,10,11,12,13]. Researchers discussed with the osteoinductive phenomenon of several ceramics from the points of adsorption of circulating BMPs on the surface, degradation products such as calcium ion (Ca^2+^), surface properties, and capillary force. However, the exact mechanism of osteoinductive process is still unknown. Recently, in vitro study reported that an increase in extracellular Ca^2+^ induced osteogenic differentiation of human adipose-derived stem cells (hASCs) by autocrine and/or paracrine signaling of BMP-2 [14]. The report indicated Ca^2+^ concentration in the pores of HAp might control to induce undifferentiated mesenchymal cells or stem cells to osteoblasts and supported a hypothesis related with degradation products such as Ca^2+^ as strongly possible mechanisms of osteoinduction [14].

### 1.2. Modification of Crystallographic Properties of Synthetic HAp

To modify crystallographic properties of synthetic hydroxyapatite (HAp), a partial dissolution-precipitation (PDP) method was originally developed by Akazawa et al. [15] for improving the bioabsorbability of sintered HAp and evolved by the supersonic demineralization for the rapid preparation [16,17]. Microcracks and trabecular fractures appear constantly in physiological bone [18]. From biological points of view, biomimetic scaffolds have been strongly needed in regenerative medicine [19,20,21]. Interconnected macropore structure is necessary to promote body fluid permeation, neovascularization, and cell migration to the central pores, and micropores can provide more nucleation sites for bone-like apatite precipitation [22,23]. The supersonic acid-demineralization produces biologically simulant microcracks on the surface and inner bulk of commercial HAp products for precipitation of nano/micro crystals [19,24]. Additionally, all biomaterials, used for bone regeneration, should enhance bone growth directly in contact with the biomaterial surface from the surrounding bone, but it should also be capable of inducing osteoinduction [25]. We speculated that the improved microstructure with large specific surface area could contribute to provide osteogenic spaces as compartment houses, and undifferentiated mesenchymal cells might recognize release of Ca^2+^ and PO_4_^3−^ from the biological apatite layer and differentiate into osteogenic cells.

The aim of this study was to observe ectopic bone formation by PDP-treated synthetic HAp block with triple-pore structure (PDP-HAp) with or without BMP-2 in the connective tissues of young rat scalp.

## 2. Materials and Methods

### 2.1. HAp Block

Commercially available HAp blocks (APACERAM AX^®^, HOYA Technosurgical Co., Tokyo, Japan) were used [26]. Briefly, the block (5 × 5 × 5 mm^3^), followed by sintering at 1200 °C, showed a triple pore-structure (porosity: 82.5%; macropore: 50–300 μm; interconnected pore: 50–100 μm; micropore: 0.5–10 μm; compression strength: 0.7 MPa).

### 2.2. Preparations of Partially Dissolved and Precipitated HAp Block (PDP-HAp)

The block was impregnated into 1.7 × 10^−2^ N HNO_3_ solutions (50 cm^3^) containing Ca^2+^ and PO_4_^3−^ and partially dissolved by the supersonic treatment at 120 W, 38 kHz, and pH 1.0 for 25 min. After adding an ammonia aqueous solution for reprecipitation, the block was matured at pH 9.0–11.0 for 24 h under a bubbling of nitrogen gas. The new HAp block was washed in distilled water and dried at 25 °C to fabricate the PDP-HAp ceramics. The conditions of PDP-method were selected, based on the previously published data of the same HAp block [19].

### 2.3. Characterization of PDP-HAp

The microstructures were observed by scanning electron microscopy (SEM). The crystalline phase was identified by micro X-ray diffraction (Micro-XRD) using CuKα_1_ radiation, and the composition ratio of Ca^2+^ to PO_4_^3−^ ion (Ca/P) was detected by electron probe microanalysis (EPMA). The porosity of the block was estimated by the water displacement method. The specific surface area was measured from N_2_-adsorption at −196 °C. For the dissolution characteristics, the dissolution efficiency was calculated from a difference in the weight of HAp before and after the supersonic treatment in 1.7 × 10^−2^ N HNO_3_ aqueous solution.

### 2.4. Implantation on Rat Skull Periosteum

**Figure 1:**
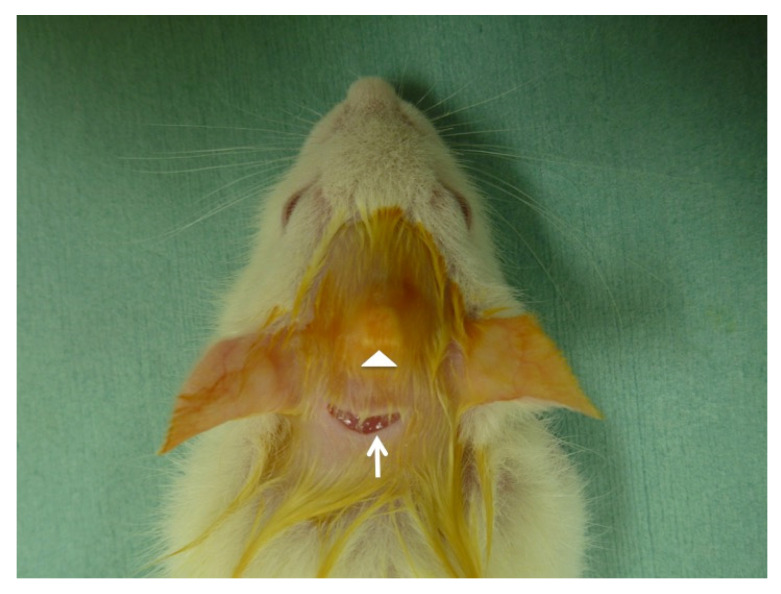
Implantation view of partially dissolved and precipitated hydroxyapatite (PDP-HAp). Arrowhead showing uplift under head skin just after implantation. Arrow indicating incision line.

**Figure 2:**
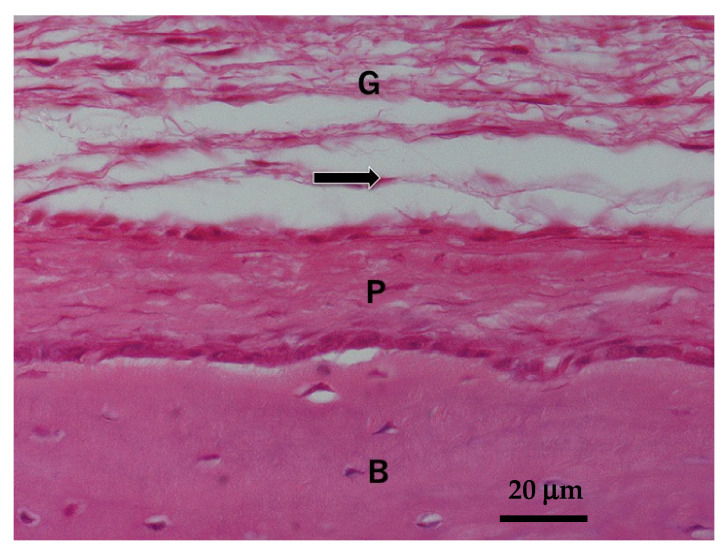
Normal skull structure of four-week-old Wistar rat. Arrow indicating implanted tissue region. B: skull bone; P: periosteum; G: galea.

Thirty rats were divided into PDP-HAp group and PDP-HAp/BMP-2 group. The PDP-HAp and the PDP-HAp/BMP-2 were implanted onto skull periosteum of Wistar rats (four-week-old, male) under general anesthesia (Figure 1). Rat galea was elevated, and each block was implanted into a space between the galea and the periosteum as a new ectopic model (Figure 2). The HAp blocks were not contacted with the calvaria bone and osteogenic cells. Then, 10 μL of BMP-2 solution (0.1 μg/μL) was added to the PDP-HAp, and 10 μL of distilled water was added to the PDP-HAp just before use. Each rat received one block. Five rats in each group were killed at 3, 6, and 9 months after the operation. This study was approved (No. 150) by the Animal Ethics Committees of Health Sciences University of Hokkaido with the principles of the Declaration of Helsinki. The experimental procedure for this study was also followed by the ARRIVE guidelines [27].

### 2.5. Histological Observations

Samples excised with scalp and skull were fixed in 10% neutral buffered formalin, decalcified, embedded in paraffin, and sectioned. The specimens were stained with hematoxylin and eosin (HE). Tissues were observed histologically by light microscopic.

### 2.6. Morphometric Analysis

Tissues in the PDP-HAp and the PDP-HAp/BMP-2 were divided into three compartments: bone, which included induced bone and marrow; HAp, which has no cellular invasion; and connective tissue, which contained collagen and fibroblasts. The compartments of the tissues were measured by using Weibel method [28] at three separate points, 40 μm apart, with the midpoint being near center of the PDP-HAp. The average value of the three points was used as the mean area of each implant. Statistical significance of the data was evaluated by Student’s *t*-test.

## 3. Results

### 3.1. SEM Micrographs of Original HAp and PDP-HAp

**Figure 3:**
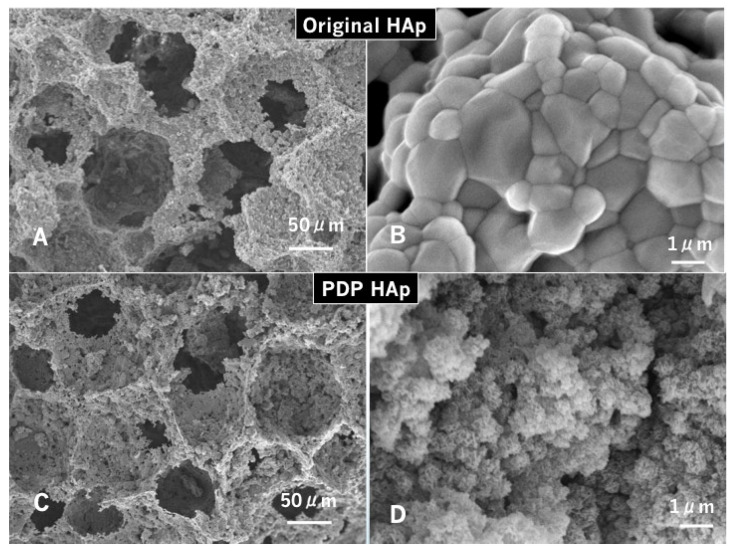
Scanning electron microscopy (SEM) photographs of original HAp (**A**,**B**) and PDP-HAp (**C**,**D**). (**A**) Interconnected pore structure. (**B**) Smooth surface. Note: uniform large grains (1.0–3.0 μm). (**C**) Irregular surface. Note: small dot holes (micropores) on macropore walls. (**D**) Spherical moss-like grains (0.5–1.0 μm) forming needle-like nano-sized crystals.

The original HAp revealed macro-pore (50–200 μm), interconnected pore, and micro-pore (0.5–10 μm), named as a three-dimensional interconnected pore structure (Figure 3A,B). The surface grains were flat and homogeneously large (1.0–3.0 μm) (Figure 3B). On the other hand, the PDP treatment dramatically changed the microstructure, and the PDP-HAp showed rough dots on the pore walls (Figure 3C). Spherical moss-like grains (0.5–1.0 μm) forming needle-like nano-crystals that were deposited onto the surface of the large grains (Figure 3D). 

### 3.2. Characterization of PDP-HAp

Micro-XRD of the PDP-HAp showed single phase of HAp, and Ca/P ratio was 1.64–1.66. The PDP-HAp exhibited 50–200 μm in the macro-pore sizes, 85–90% in the porosity, and 1.0–2.0 m^2^/g in the specific surface area.

### 3.3. Histological Findings

**Figure 4:**
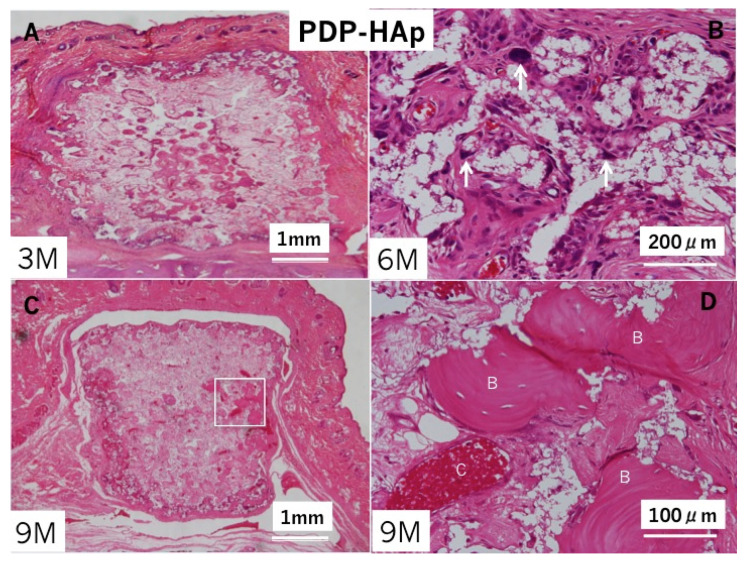
Histological photographs of HE sections in PDP-HAp alone. (**A**) Tissue formation in pores at three months. (**B**) None of bone and cartilage at six months. Arrow: giant cells. (**C**) Rectangle indicating bone induction at nine months. (**D**) Higher magnification of rectangle in (**C**). Induced bone in micro-pores at nine months.

In the PDP-HAp group, fibrous connective tissues were observed in the peripheral pores of the block at three months (Figure 4A). Multinucleated giant cells appeared in the surrounding pore-wall areas at 6 months (Figure 4B). Spindle-type mesenchymal cells were seen with capillary in the central pore areas at six and nine months (Figure 4C). Bone induction was found at nine months in the pores, and cement lines were observed in the induced bone (Figure 4D). Implanted PDP-HAp block was never connected with the original skull.

**Figure 5:**
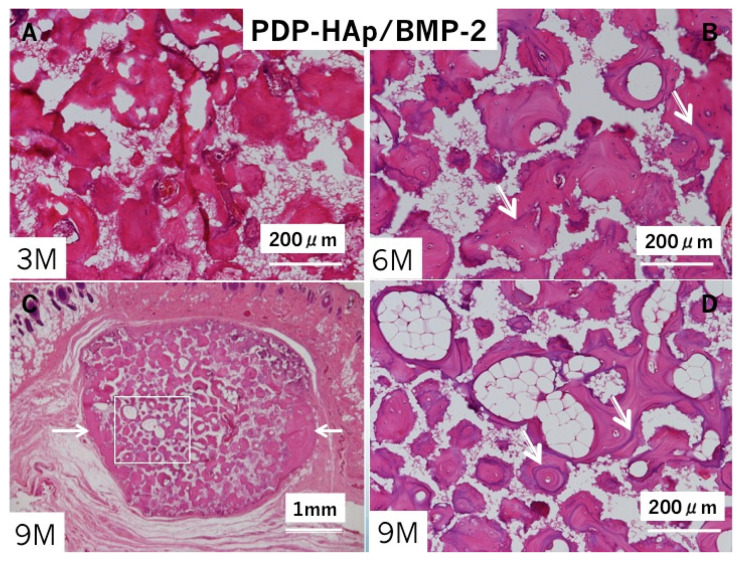
Histological photographs of HE sections in PDP-HAp/BMP-2. (**A**) Bone induction in macro/micro-pores at three months. Note: no cartilage. (**B**) Bone induction in all pores at six months. Arrows: remodeling lines. (**C**) Whole appearance at nine months. Bone induction in pores and surroundings of PDP-HAp. Arrow indicating dense bone area. (**D**) Higher magnification of rectangle in (**C**). Induced bone with fatty marrow in interconnected pores at nine months. Arrows: remodeling lines.

In the PDP-HAp/BMP-2 group, bone induction occurred inside almost all pores at three months (Figure 5A). Bone accompanied with marrow cells. Multinucleated giant cells appeared especially in the surrounding areas of the block. Cement lines in the induced bone were observed clearly at 6 months (Figure 5B). Bone with fatty marrow was found mainly in the central pore area, and bone at the surrounding area developed like cortical bone at nine months (Figure 5C,D). Fibrous connective tissues existed between the PDP-HAp/BMP-2-induced bone and periosteal tissues. Induced bone was never connected with the skull (Figure 5C). The HAp residues (white clear space without cells) decreased in the induced bone area showing cortical bone structure (Figure 5C).

### 3.4. Morphometric Findings

The morphometric results are shown in Table 1. The PDP-HAp alone implant showed 0.0% at three months in the volume of bone tissue, while the PDP-HAp/BMP-2 implant showed 77.0% at three months. At nine months, the PDP-HAp alone showed 12.0% in the bone volume, while the PDP-HAp/BMP-2 showed 86.0%. The ratio of volume of bone in the PDP-HAp at nine months was 14.0% of that in the PDP-HAp/BMP-2.

## 4. Discussion

Bone induction in the connective tissues of young rats’ scalps was found for the first time in the PDP-HAp block. Until now, several porous ceramics induced ectopic bone in the muscles of large animals such as dog, sheep, goat and baboons at 1–6 months after implantation [1,2,3,4]. Subcutaneous implantation of several porous ceramics did not give rise to bone induction in goats, while bone was induced intramuscularly in most goats [4].

Briefly, the same ceramics induced bone intramuscularly but not subcutaneously. Several groups have suggested the importance of both vascularization and micropores with increased specific surface area in the process of osteoinduction [4,10,11,12,13]. Based on the previous reports, a bioassay in the connective tissue model has not a better condition in ectopic bone formation than that in the muscles. It is well known that vascularization in the connective tissues is less than that in the muscles. In this study, the PDP-HAp alone induced bone in non-osteogenic connective tissues of rat scalp at nine months. In contrast, the PDP-HAp/BMP-2 (1.0 μg) induced bone at two weeks (data not shown). Generally, bone and cartilage were induced independently at two weeks by porous HAp with BMP-2 (1.0 μg) [22,26]. In the BMP-loaded systems, BMP-2 molecule can differentiate mesenchymal cells into osteoblasts not only on flat plate of ceramics, but also on organic scaffolds at 1–2 weeks. BMP-2 can induce angiogenesis and osteogenesis in and/or on ceramic- and composite-based materials [22,29]. The bone-inductive speed of the PDP-HAp was completely different from the PDP-HAp/BMP-2 in this model. Therefore, the osteoinduction in the micropores of ceramic-based materials such as PDP-HAp may be a different mechanism from the BMP-loaded HAp-induced bone formation.

From a point of bone storage views, it is easy to take out materials and/or BMP-induced bone tissues from the position between the galea and the periosteum. Implanted materials and/or BMP-induced bone didn’t unite skull in the present study. If biomaterials were implanted into an osteogenic space between periosteum and skull, the biomaterials could be connected directly with skull as vertical bone augmentation like onlay model [30]. Our chosen location may be a unique soft tissue environment for research works and graft bone storage for clinical uses.

Our highly porous PDP-HAp should be more degradable than original HAp in living body. As for the characterization of PDP-HAp, microcracks inside the HAp bulk formed by the stirring-supersonic treatment in 2% HNO_3_ would result in increases of surface area for CaP precipitation and body fluid permeation inside the bulk, and then enhance ionic degradation in micropores. In vivo implantation of PDP-HAp used in this study revealed that PDP-HAp was gradually degraded and was replaced by new bone in the rabbit bone defect model [31]. Moreover, in vitro osteoclastogenesis assay resulted in significant larger TRAP-staining area and higher expression of osteoclast-related genes in the PDP-HAp than in the dense HAp [31]. Biological crystals like PDP layer should be a mineral signal in the recruitment and differentiation of multinucleated giant cells. Stem cells near capillary in micropores might recognize the biological apatite layer with proteins, the growth factors released from multinucleated giant cells, and the flow potential by both the dissolution-reprecipitation of ions and the cell movement as signals. In cellular environments, changes in intracellular calcium level ([Ca^2+^]_in_) act as a universal signal that intersects with many pathways regulating gene expression [32], and the elevated [Ca^2+^]_in_ induces chondrogenesis of mesenchymal cells by stimulating BMP-2 expression via a calcineurin/nuclear factor of activated T-cells (NFAT) pathway [33]. Additionally, the expression of BMP-2 of hASCs was enhanced by an elevation of extracellular calcium level via activation of a calcium-sensing receptor (CaSR) and recruitment of Ca^2+^/calmodulin-dependent NFAT-signaling pathways [14]. Briefly, extracellular Ca^2+^ can stimulate osteogenic differentiation of hASCs by enhancing BMP-2 expression. Based on the above in vitro evidences, we speculated that undifferentiated mesenchymal cells near blood vessels in microporosity recognized the elevation of intercellular Ca^2+^ release from the biological PDP layer in micropores, enhanced the expression of BMP-2 and then differentiated into osteoblasts in the pores. An osteoinductive biomaterial should be able to recruit osteoprogenitor cells and transform undifferentiated mesenchymal cells into osteoblasts [34]. The cascade of biological events in bioceramics-induced osteogenesis will become clear in the near future.

## 5. Conclusions

The PDP method contributed to the modification of the originally synthetic HAp as for the microcrack formation inside bulk and the increase of specific surface area by biological HAp layer. Bone induction was observed in micropores of the PDP-HAp block, implanted into a unique space between galea and periosteum of rat at nine months. The results indicated the PDP-HAp showed osteogenic microporous compartments with partially absorbable HAp crystals.

## Figures and Tables

**Table 1:** The changes in proportion (%) of each tissue to total volume of implant.

Groups	Bone	HAp	CT
PDP-HAp 3 M	0.0 ± 0.0 *	15.0 ± 1.7	85.0 ± 1.7
PDP-HAp 9 M	12.0 ± 5.3 **	13.0 ± 2.1	75.0 ± 3.7
PDP-HAp/BMP-2 3 M	77.0 ± 2.1 *	14.0 ± 0.5	9.0 ± 1.7
PDP-HAp/BMP-2 9 M	86.0 ± 1.4 **	11.0 ± 0.8	3.0 ± 0.8

The total volume is designed as 100%. Bone: bone with marrow; HAp: hydroxyapatite; CT: connective tissues. Values are mean ± standard deviation (SD). Significant differences: *, ** = *p* < 0.01, *N* = 3.

## Data Availability

Data sharing is not applicable to this article.
